# Cultivation of the polyextremophile *Cyanidioschyzon merolae* 10D during summer conditions on the coast of the Red Sea and its adaptation to hypersaline sea water

**DOI:** 10.3389/fmicb.2023.1157151

**Published:** 2023-04-20

**Authors:** Melany Villegas-Valencia, Ricardo E. González-Portela, Bárbara Bastos de Freitas, Abdulaziz Al Jahdali, Gabriel I. Romero-Villegas, Raghdah Malibari, Rahul Vijay Kapoore, Claudio Fuentes-Grünewald, Kyle J. Lauersen

**Affiliations:** ^1^Bioengineering Program, Biological and Environmental Sciences and Engineering Division, King Abdullah University of Science and Technology (KAUST), Thuwal, Saudi Arabia; ^2^Development of Algal Biotechnology in Kingdom of Saudi Arabia (DAB-KSA) Project, Beacon Development, King Abdullah University of Science and Technology (KAUST), Thuwal, Saudi Arabia

**Keywords:** circular resource biotechnology, desert, *Cyanidioschyzon merolae* 10D, sea water, Saudi Arabia Red Sea coast, algal biotechnology

## Abstract

The west coast of the Arabian Peninsula borders the Red Sea, a water body which maintains high average temperatures and increased salinity compared to other seas or oceans. This geography has many resources which could be used to support algal biotechnology efforts in bio-resource circularity. However, summer conditions in this region may exceed the temperature tolerance of most currently cultivated microalgae. The Cyanidiophyceae are a class of polyextremophilic red algae that natively inhabit acidic hot springs. *C. merolae* 10D has recently emerged as an interesting model organism capable of high-cell density cultivation on pure CO_2_ with optimal growth at elevated temperatures and acidic pH. *C. merolae* biomass has an interesting macromolecular composition, is protein rich, and contains valuable bio-products like heat-stable phycocyanin, carotenoids, β-glucan, and starch. Here, photobioreactors were used to model *C. merolae* 10D growth performance in simulated environmental conditions of the mid-Red Sea coast across four seasons, it was then grown at various scales outdoors in Thuwal, Saudi Arabia during the Summer of 2022. We show that *C. merolae* 10D is amenable to cultivation with industrial-grade nutrient and CO_2_ inputs outdoors in this location and that its biomass is relatively constant in biochemical composition across culture conditions. We also show the adaptation of *C. merolae* 10D to high salinity levels of those found in Red Sea waters and conducted further modeled cultivations in nutrient enriched local sea water. It was determined that salt-water adapted *C. merolae* 10D could be cultivated with reduced nutrient inputs in local conditions. The results presented here indicate this may be a promising alternative species for algal bioprocesses in outdoor conditions in extreme coastal desert summer environments.

## 1. Introduction

Currently, bioprocesses that use microalgae are being implemented with increasing intensity in nearly all countries due to their broad potential for low input waste-revalorization (Vigani, 2020). Algae can convert the nutrients and trace elements and CO_2_ from waste streams into their biomass using light energy *via* photosynthesis (Fuentes-Grünewald et al., 2012). There are numerous market opportunities for different products made from algal biomass such as animal feed, food, pigments, bioplastics, speciality chemicals, and biostimulant fertilizers (Rumin et al., 2020). The worldwide production of microalgal biomass is currently low, ~56,000 tons *per annum* compared to production of marine seaweeds, ~34.6 M tons *per annum* (Ferdouse et al., 2018; Cai et al., 2021). There is currently an opportunity, especially in desert countries, for increased microalgal production processes aimed at bio-resource circularity, especially connected to urban and industrial areas (Lefers et al., 2020; Greene et al., 2022).

Algae can be cultivated in inexpensive photobioreactors built on non-arable land and directly connected to industrial side-streams to derive value from effluents produced in otherwise overlooked geographical areas such as desert regions or other extreme environments (Fuentes-Grünewald et al., 2012; Vigani, 2020; Cabrera-González et al., 2022). The implementation of algal bioprocesses in emerging human settlement contexts will further promote algal biomass and bioproduct integration into common use in the bioeconomy (Cabrera-González et al., 2022). Microalgal bioprocesses are most strategically suited to geographies with high solar irradiation and stable temperatures, having local CO_2_ emissions sources, non-arable land, access to sea water, and inexpensive energy sources (Fuentes-Grünewald et al., 2012; Greene et al., 2022; Schipper et al., 2022). Most desert countries have coastal regions, including the Arabian Peninsula, Northern Australia, Chile, and North Africa (Lefers et al., 2020; Greene et al., 2022). At these locales, saline microalgal processes are logical (Ishika et al., 2017), and inland, where human settlements occur, freshwater algal cultivation can participate in waste-water treatment and reuse (de Freitas et al., 2023). It was recently reported that the geographical areas with the highest potential for onshore marine microalgae biomass production were between 30°N and 30°S in the so-called “hot belt countries” (Greene et al., 2022). This area is dominated by countries with enormous desert regions such as the southern US (Arizona), Northern Chile (Atacama), Northern Africa, Northern Australia, and the Middle East. It is predicted that in these environments, microalgal biomass production from local inputs can be maximized (Greene et al., 2022). To capture the value of resources in waste-streams, the development and integration of algal bioprocesses can play a role in circular reuse concepts in these environments (Cabrera-González et al., 2022). New target microalgal species with high biotechnological potential, that can tolerate extreme conditions must be identified, characterized, and developed for these contexts. Some work has already been performed to bioprospect species from these environments (Barten et al., 2020; Alshareef, 2021; Almutairi, 2022; Schipper et al., 2022), however, little has translated to application in algal cultivation concepts, especially in the Middle East.

The unicellular red microalga *Cyanidioschyzon merolae* is a poly-extremophile that thrives in low pH and high temperatures. *Cyanidioschyzon merolae* 10D was originally isolated from a sample taken from the Phelgrean fields near Naples, Italy (Matsuzaki et al., 2004). Its biomass contains interesting amounts of valuable metabolites such as thermostable phycocyanin (PC) in addition to starch, β-glucan, β-carotene and zeaxanthin carotenoid pigments (Cunningham et al., 2007; Miyagishima and Tanaka, 2021). This extremophile exhibits its best growth at temperatures from 42 to 50°C and low pH (0.5–2.5) (Miyagishima and Tanaka, 2021). *C. merolae* 10D was the first eukaryotic algae to have a telomere to telomere sequenced genome and is capable of homologous recombination in its nuclear genome (Matsuzaki et al., 2004; Fujiwara et al., 2013, 2017, 2021). It is a highly useful model organism for cellular studies, containing only one of every organelle and rapid generation times (Miyagishima and Tanaka, 2021). A recent report has also shown it is possible to metabolically engineer this organism for alternative ketocarotenoid production (Seger et al., 2023). Tolerance to acidic conditions can allow cultivation with continuous injection rates of high concentration CO_2_ and ammonia while also minimizing contamination (Lang et al., 2020). *Cyanidioschyzon merolae* 10D has also previously been shown amenable to adaptation to saline conditions, suggesting it may be a flexible host for sea-water based algal biotechnology in coastal routinely high temperature environments (Hirooka et al., 2020). This species may be a promising candidate for algal bioprocesses in extreme environments and may also be a host with future value in synthetic biology and genetic engineering concepts. *Cyanidioschyzon merolae* 10D is an excellent candidate for waste circularization concepts which seek to take high concentration feed streams, especially in summertime conditions in desert environments, and convert them into valuable biomass (Lang et al., 2020).

In order to conduct algal waste-stream recovery in unfavorable, hot climates extremeophiles which are not currently standard species in algal cultivation concepts must be benchmarked. We sought to test whether a known polyextremophile with promising phenotypic and genetic properties would perform favorable under mid-Red Sea coastal conditions in the mid summer. Here, we show the performance characteristics of *C. merolae* 10D in lab-scale 400 mL Algem photobioreactors using cultivation programs to emulate different seasons on the mid Red Sea coast in Saudi Arabia, successful scaling of these cultures to outdoor pilot scale (1 m^3^) photobioreactors in summer desert conditions, biomass characterization, and the adaptation of this species to high saline conditions to allow cultivation with medium made using Red Sea water with average salinity of 42+2%. This is the first report that *C. merolae* 10D has been grown at a pilot scale and the first to demonstrate its potential use for industrial applications in desert contexts. Our results indicate that *C. merolae* 10D, and maybe other Cyanidiophyceae, will be valuable for bioproduction of products such as proteins, oils, and pigments or waste-stream bio-conversion in summer conditions in extreme desert environments where other common commercial algal species may exhibit temperature threshold limitations.

## 2. Materials and methods

### 2.1. Lab-scale cultivation of *Cyanidioschyzon merolae* 10D and bioreactor growth tests

*Cyanidioschyzon merolae* 10D (Toda et al., 1995; Matsuzaki et al., 2004) was received from the lab of Prof. Peter Lammers (Arizona Center for Algae Technology Innovation (AzCATI), Arizona State University (ASU)) which we routinely maintained in MA2 liquid medium (Minoda et al., 2004): pH 2.3, adjusted with H_2_SO_4_ in 125 mL Erlenmeyer flasks shaken at 100 rpm under 90 μmol photon m^−2^ s^−1^ (hereafter, *μ*E) continuous white light and 42°C in a Percival algae incubator supplemented with 4% CO_2_ in air mixtures. Preculturing prior to growth analysis was performed by inoculation of cells to shake flasks with MA2 medium for 96 h, then *C. merolae* 10D cells were resuspended according to desired densities in 400 mL flasks prior to growth analysis in Algem photobioreactors (Algenuity©, United Kingdom). Cultures in biological duplicates with starting cell concentrations of 3×10^6^ cells mL^−1^ were cultivated in environmental simulations of the mid Red Sea coast conditions of Thuwal, Saudi Arabia (22.3046 N, 39.1022E) as recently reported (De Freitas et al., 2023) and compared with control cultures using 12:12 h light:dark or 24 h constant 1,500 *μ*E illumination at 42°C. All cultures received continuous 7% CO_2_ in air at a 25 mL min^−1^ flow rate. Modeled environmental conditions were from each season with local historical weather data used to build profiles of temperature and light for February (Winter), May (Spring), August (Summer), and November (Autumn). Daily, 15 mL of algae culture from each replicate flask was taken for cell concentration (100 μL), and biomass quantification (3× 4 mL). Cell densities were measured using an Invitrogen Attune NxT flow cytometer (Thermo Fisher Scientific, United Kingdom) equipped with a Cytkick microtiter plate autosampler unit. Each *C. merolae* 10D biological replicate was diluted 1:100 with 0.9% NaCl solution and loaded into a 96-well microtiter plate in technical duplicates prior to injection into the flow cytometer using the autosampler. Samples were mixed three times immediately before analysis, and the first 25 μL of the sample was discarded to ensure a stable cell flow rate during measurement. For data acquisition, 50 μL from each well was analyzed. Optical densities were measured as absorbance at 740 nm (OD _740nm_) by the Algem photobioreactors every 10 min. Cell dry mass was measured by vacuum filtration of 3× 4 mL of each biological replicate on pre-weighted filters (0.45 μm). The algal cell masses were dried at 60°C for 24 h prior to weighing.

To adapt *C. merolae* 10D to saline conditions and enable its cultivation on a medium made from acidified seawater, MA2 medium was prepared with different dilutions of filtered and autoclaved Red Sea water (RSW-MA2) as 100, 80, 60, 40 and 20% mixtures with fresh water plus MA2 nutrients. In a 6-well plate, 200 μL *C. merolae* 10D late logarithmic phase preculture culture was inoculated into wells each containing the different RSW-MA2 concentrations. The cells were cultivated in the algal incubator for 168 h as above. Cultures which reached reasonable cell densities were re-inoculated in new media across the salinity dilution range and grown for another 168 h. This process was repeated another two times and the culture adapted to 100% RSW-MA2 was chosen for further cultivation in emulated local weather conditions in Algem photobioreactors as described above.

### 2.2. Cultivation of *Cyanidioschyzon merolae* outdoors at increasing culture volumes

Precultures of *C. merolae* 10D grown in Algem photobioreactors were pooled and transported to the Development of Algal Biotechnology Kingdom of Saudi Arabia (DAB-KSA) pilot plant (KAUST, Thuwal, Saudi Arabia), and cultivated outdoors in various photobioreactors from June–August 2022. Cultures were started in 8 L south-facing glass columns at outdoor conditions with 39 ± 4.0°C and mid-day irradiance 1,503 ± 241 *μ*E, for 2 weeks. The culture medium was prepared using Altakamul industrial/agricultural-grade salts (ammonium sulfate and mono-potassium phosphate–purity of 89.95 and 92.25%, respectively). The medium contained 5.3 g (NH₄)₂SO₄, 1.09 g KH₂PO₄ and 0.481 g MgSO_4_ L^−1^ with F/2 medium trace elements added to 1 mL L^−1^ (Guillard, 1975). The pH was adjusted to 2.5 with H_2_SO_4_. Additionally, an ammonium-based booster solution (10 mL L^−1^, with 50% of the original initial concentration of NH_4_^+^) was added every 48 h during the whole cultivation period. Once enough inoculum was produced, the culture was transferred to a 60 L column photobioreactor (Varicon Aqua® Phyco-Bubble model–column of 20 cm of diameter and 2.25 cm of height, and working volume of 60 L) with constant CO_2_ sparging from the bottom at a rate of 0.2 L min^−1^ and the unit was exposed to outdoor conditions of 32.4 ± 2°C, 1351 ± 110 *μ*E. This culture was used as an inoculum for a 600 L raceway (Varicon Aqua® Phyco-Pond model) for 1 week, and then it was transferred to a 1,000 L tubular photobioreactor (PBR, Varicon Aqua® Phyco-Flow model) and the cultivation run for 576 h. Finally, this culture was used as inoculum for two other 1,000 L PBRs started at 30% dilution from the first reactor. Two reactors were run with 100% CO_2_ sparging–PBR-1: “Commercial” pure CO_2_ Alpha Gaz®, 99.995% (CG) and PBR-2: “Green” CO_2_ from flue gas, Gulf Cryo® (GC), while a third was not given CO_2_–PBR-3: “Air” 0.04% CO_2_ (AC). The three reactors are labeled PBR 1–CO_2_, PBR 2–CO_2_, and PBR 3–Air. *C. merolae* can tolerate pure CO_2_ injections at high rates due to the acidic preferences of this alga (Lang et al., 2020; Miyagishima and Tanaka, 2021). In all treatments, gassing was supplied constantly (0.2 L min^−1^) from 8 am to 5 pm as it would not be consumed during non-illuminated times.

OD _750nm_ was recorded daily with a UV–Vis Spectrophotometer (Thermo Scientific® Genesys™ 50, United Kingdom). Cell dry weight measurement (in technical duplicates) was performed every other day using a gravimetric method, where aliquots were rinsed with acidified fresh water (pH 2.5) and filtered through glass microfiber filters (Whatman GF/F) and weighed after drying. Nutrient uptake was measured as N (SPECTROQUANT® Ammonium Kit 1.00683.0001) and P (SPECTROQUANT® Phosphate Kit 1.00798.001), using Spectroquant® assay kits following manufacturer’s protocols. Temperature and pH were monitored continuously during the experiment (10 days) with the built-in probes of the PBRs. Light intensity was measured as irradiance (quantum flux) with a quantum meter probe (Biospherical instruments Inc.® Model QSL2000). For biochemical characterizations, at the incubation site, the cell suspension was harvested and washed twice with acidified freshwater (pH 2.5). The washed microalgal pellets were freeze-dried overnight and stored in dark at−20°C until further analysis.

### 2.3. Biochemical characterization of *Cyanidioschyzon merolae* 10D biomass

All chemicals and analytical reagents were HPLC grade (Fisher Scientific, United Kigdom) unless stated otherwise. A multi-assay procedure (Chen and Vaidyanathan, 2012, 2013; Kapoore et al., 2019) was modified for the quantification of total protein, carbohydrate, chlorophyll and carotenoids as follows. Briefly, freeze-dried microalgal pellets were weighed (1.5–2 mg) in 2 mL Safe-Lock microcentrifuge tubes. The dry pellets were resuspended in 24.3 μL of phosphate Buffer (pH 7.4) and 1.8 mL of 25% (*v/v*) methanol in 1 M NaOH along with an equal volume of glass beads (425–600 μm i.d., acid washed). Cells were disrupted using Retsch MM 400 Mixer Mill (Retsch, GmbH) for 3 cycles (10-min bead beating and 2-min stand). For carbohydrate analysis, 2× 0.2 mL extract was transferred to 2 mL PTFE capped glass vials: for the control by adding 1.2 mL 75% H_2_SO_4_; for the experimental sample by adding 0.4 mL 75% H_2_SO_4_ and 0.8 mL anthrone reagent; completing the assay as described (Chen and Vaidyanathan, 2012, 2013; Fuentes-Grünewald et al., 2012; Kapoore et al., 2019) by incubating at 100°C for 15 min followed by measurement in polystyrene cuvettes (ΔA_578_). The remainder extract after cell disruption was stored at −80°C in 4 mL PTFE capped glass vials and later saponified as described (Chen and Vaidyanathan, 2012, 2013; Kapoore et al., 2019; Fuentes-Grünewald et al., 2012) by incubating at 100°C for 30 min. The saponification treatment promotes the release of cellular proteins and fully extracts all the proteins. In addition, the structural transformation of chlorophylls caused by saponification leads them to separate from the family of fat-soluble pigments (Chen and Vaidyanathan, 2013). As a result, the total pigments are segregated into two parts: water-soluble chlorophylls in the aqueous phase and fat-soluble carotenoids in the organic phase. After saponification, the remainder extract is then used in the subsequent pigment and protein analysis. For the pigment assay, saponified extracts of 0.7 mL were transferred after vortexing to 2 mL Safe-Lock microcentrifuge tubes containing 1.05 mL 2:1 mixture of chloroform with methanol. After centrifugation and phase separation, chlorophyll and carotenoid concentrations were determined as previously described (Chen and Vaidyanathan, 2012, 2013; Kapoore et al., 2019). The remainder of the saponified extract was stored at −80°C for subsequent protein assay where saponified extracts of 25 μL were first placed directly into 96-well assay plates with the following additions: controls, 0.2 mL BCA reagent alone (Thermo Scientific); experimental, 0.2 mL BCA/Cu mix (Thermo Scientific) and incubated at 37°C for 30 min, measuring ΔA_562_.

A previously reported procedure for phycocyanin extraction was modified for the assessment of this pigment in *C. merolae* 10D (Coward et al., 2016). Briefly, 1 mL of microalgal cell suspension was harvested and then centrifuged for 5 min at 2,500 × *g* at 4°C supernatant was decanted and the biomass was washed X3 with 1 mL of acidified water (pH 2.5). After washing, 1 mL of water was added to the wet biomass followed by four consecutive freezing (−20°C) and thawing (4°C) cycles for cell disruption. The extract was vortexed and then centrifuged at 10,000 × *g* for 10 min to remove cell debris. The clear blue supernatant containing extracted phycocyanin was quantified using spectrophotometry with OD at 455, 564, 592, 618 and 645 nm using GENESYS 50 UV–Vis Spectrophotometer (Thermo Fisher Scientific). The concentrations of phycocyanin were quantified using the previously reported equation (Coward et al., 2016).


$$\begin{array}{c} PC\;(mg\;mL^{-1})=\left[(OD_{618nm}-OD_{645nm})-(OD_{592nm}-OD_{645nm})\times 0.51\right]\\ \times 0.15 \end{array}$$

## 3. Results

### 3.1. Modeled growth of *Cyanidioschyzon merolae* 10D in lab-scale photobioreactors

**Figure 1:**
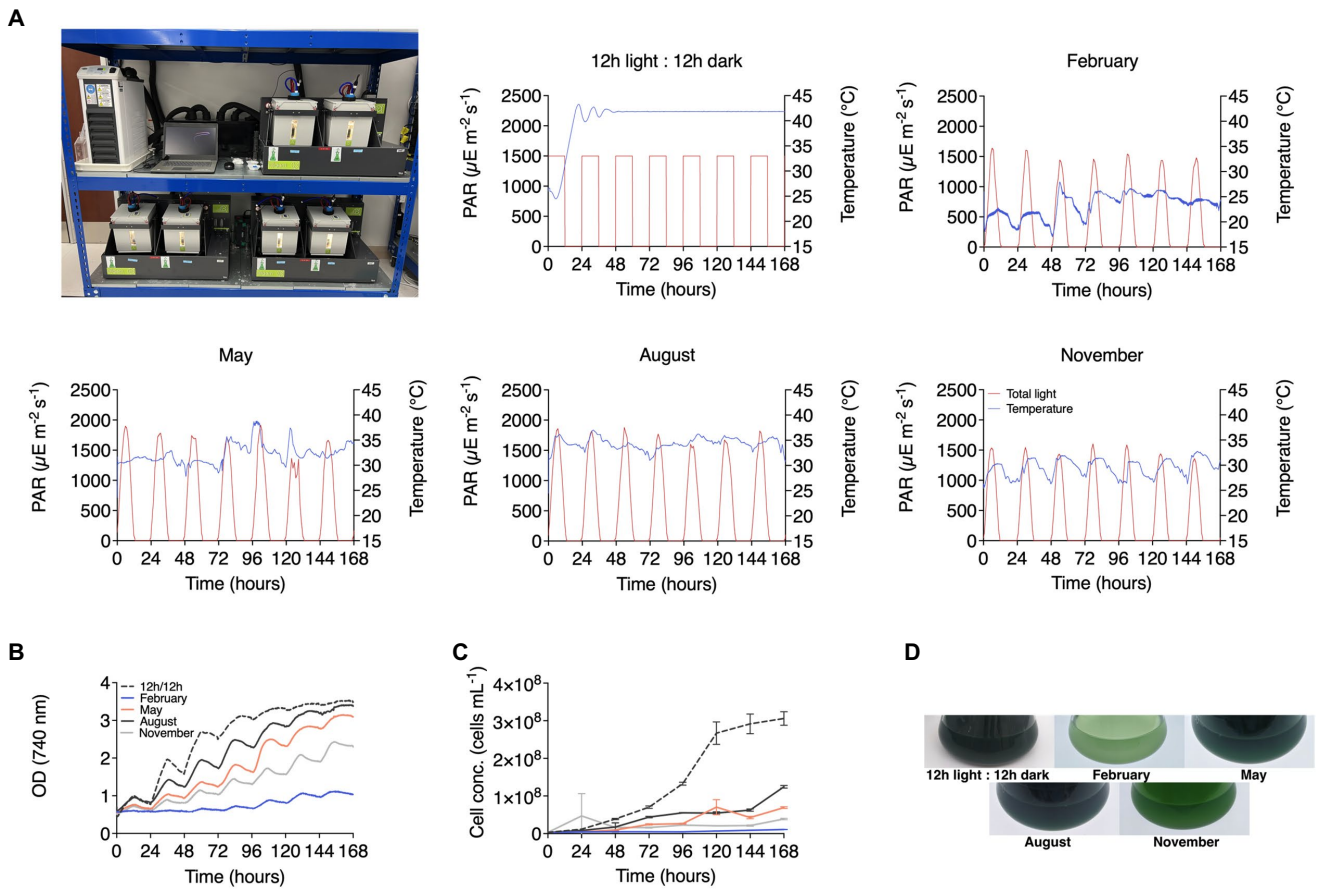
Modeled seasonal productivity variation of *C. merolae* 10D in photobioreactors. **(A)** Temperature and light profiles in Algem photobioreactors (pictured) which are based on historical weather data from Thuwal, Saudi Arabia were used to cultivate *C. merolae* 10D in MA2 medium and assess performance across different months in this locale. A control culture was set to 42°C with 12:12 h day:night illumination cycling, while February, May, August, and November profiles were used to model winter, spring, summer, and autumn, respectively. Growth performance was assessed by optical density **(B)** recorded every 10 min in the bioreactors and cell densities **(C)** which were recorded daily by flow cytometry. **(D)** Culture health and densities were also assessed photographically at the end of cultivations.

The Cyanidiophyceae tolerate extreme conditions in their native environments on the edges of acidic hot springs. Here, we modeled whether the extreme conditions *C. merolae* 10D has evolved to thrive in would enable its cultivation in conditions which are found at the mid-Red Sea Saudi Arabian coast and by extension to similar desert countries in different seasons. Photobioreactor programs were designed using local weather-station data to set light and temperature profiles in lab-scale photobioreactors (de Freitas et al., 2023) and cultures were grown with standard laboratory MA2 medium (Minoda et al., 2004). The individual seasonal conditions, Winter–February, Spring–May, Summer–August, Autumn–November, were compared to a control culture grown with a set light cycle like the photoperiod found in Saudi Arabia (12,12 day:night), and constant temperature of 42°C (Figure 1A). Culture performance was determined by optical (Figure 1B) and cell densities (Figure 1C) throughout the 192-h cultivation. Photographs of the cultures are also shown to indicate culture density and apparent health at the end of cultivation using the different modeled seasons (Figure 1D). The best culture performance was observed in the control culture with constant temperature and OD_740nm_ above 2 were achieved fastest in Summer, Spring and Autumn reactor programs, respectively (Figure 1B).

### 3.2. Scaled cultivation of *Cyanidioschyzon merolae* 10D outdoors on the mid-Red Sea coast

**Figure 2:**
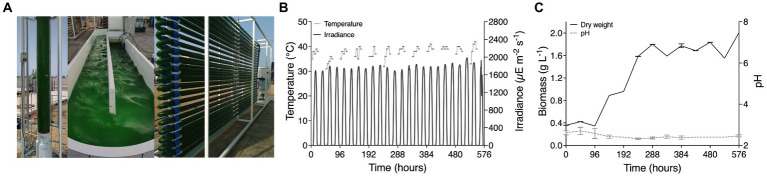
Scaled cultivation of *C. merolae* 10D in various culture set-ups in Thuwal, Saudi Arabia in July and August 2022. **(A)** Cultures in a 60 L column photobioreactor were used as inoculum for a 600 L raceway pond, which was consequently used to inoculate a 1 m^3^ tubular photobioreactor (picture left). **(B)** Daily irradiance at the tubular reactor and temperature, every other day, for 576 h during cultivation (middle). **(C)** Culture performance was measured by cell dry weight throughout the cultivation and pH was also recorded (right).

From May until August 2022, scaled cultivations of *C. merolae* 10D were performed outdoors using natural light irradiance and temperature at the DAB-KSA algal pilot facility at KAUST. Cultures were taken from lab-scale cultivations above, inoculated in 8-L columns and grown in medium prepared with industrial grade fertilizers as described in the Material and Methods section. From 8-L columns (6-L working volume), *C. merolae* 10D inoculum was transferred to a 60 L vertical column with pure CO_2_ injection during daylight hours and subsequently to a 600 L raceway pond over several weeks (Figure 2). In August, the raceway pond culture was used as inoculum for starting a 1,000 L tubular photobioreactor (at 0.38 g L^−1^) culture, which was maintained for 576 h of operation in batch mode (Figure 2). The culture experienced temperatures between 32 and 42°C (average 37.7°C) and daily peak irradiance between ~1800–1980 *μE* (Figure 2B). After the 120 h lag phase, the culture exhibited a steady increase in biomass for 168 h reaching a maximum biomass production of 1.8 g L^−1^ with 288 h of cultivation (Figure 2). The maximal productivity achieved was ~300 mg L^−1^ d^−1^ between 96 and 288 h of the cultivation.

**Figure 3:**
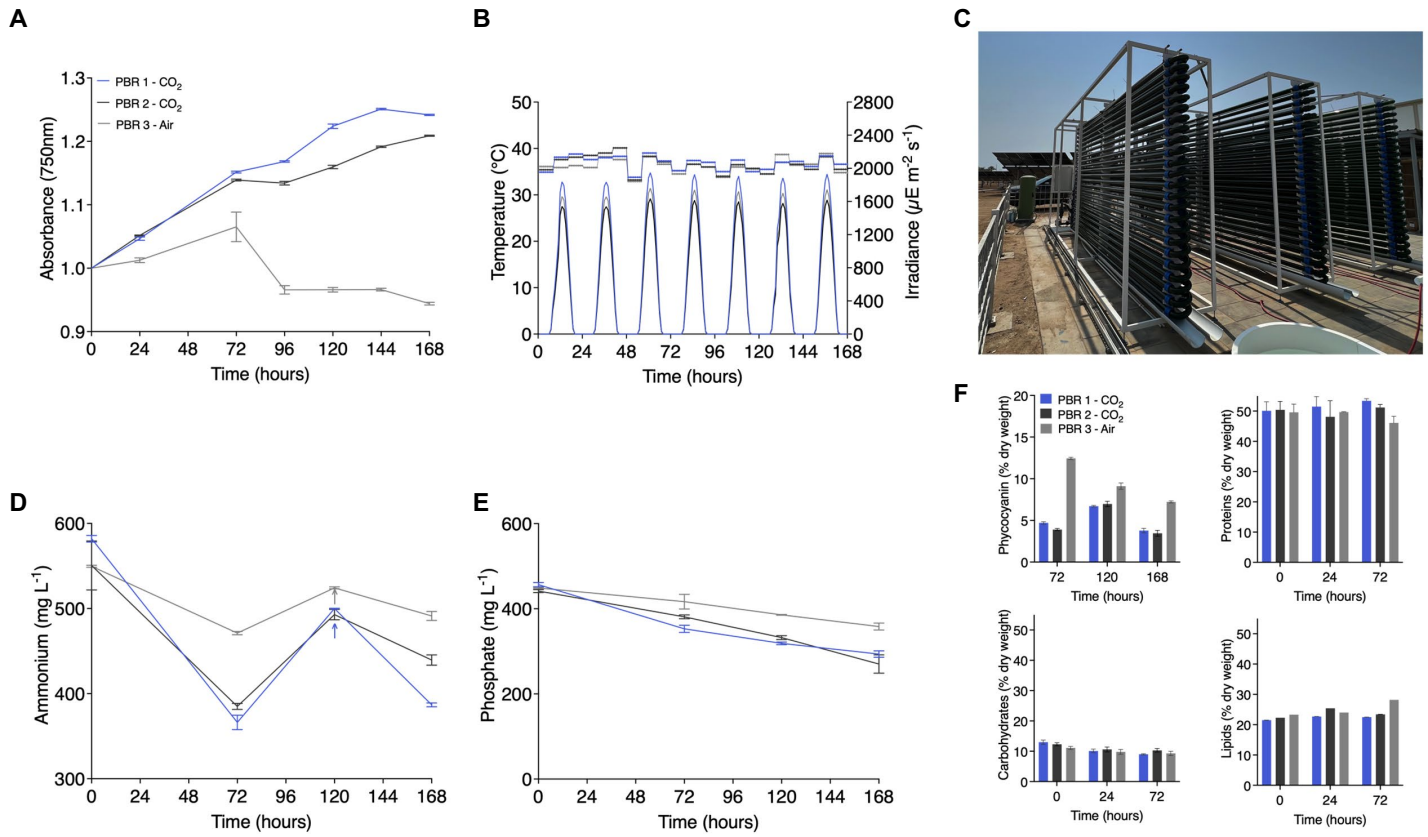
Three 1 m^3^ tubular photobioreactors were inoculated with the stationary phase culture of Figure 2. **(A)** OD_750nm_, **(B)** temperature, and irradiance were measured throughout the cultivation. The three reactors **(C)** were given beverage grade (PBR 1–CO_2_), Industrially reclaimed “green” CO_2_ (PBR 2–CO_2_), or atmospheric air (PBR 3–Air). The reactors each experienced slightly different irradiances based on their position to one another, with the outermost having higher incident light intensities. **(D)** Ammonium and **(E)** Phosphate levels were recorded at different intervals, and supplemental ammonium solution was added to all reactors after 120 h. **(F)** Culture biomass was analyzed at different time-points for phycocyanin, protein, carbohydrate, and lipids content.

The biomass obtained from this 1,000 L tubular reactor was used as inoculum for two other reactors set beside the first (Figure 3). The inoculum allowed each reactor to be started with OD 1.0 and each reactor was cultivated for an additional 144 h (Figure 3). The reactor position determined maximal light irradiance, with the middle reactor receiving less light than the external reactors (Figure 3). Culture temperatures were relatively consistent across the 3 units, between 34 and 40°C (average 36.5°C) (Figure 3). One outer (east facing, PBR 1–CO_2_) and inner reactor (PBR 2–CO_2_) were cultivated with pure CO_2_ injections while one outer (west facing, PBR 3–Air) reactor was only sparged with air. As anticipated, the air cultivated culture (PBR 3–Air) did not proliferate beyond inoculum density, while those given CO_2_ injections (PBR 1&2–CO_2_) continued to increase in their optical densities (Figure 3). These behaviors mirrored ammonium and phosphate uptake rates, which consistently was taken up by *C. merolae* cells during the cultivation period. At ~120 h, supplemental ammonium was added to the cultures to determine if the extremophile could continue to consume this in excess, and a second boost in terms of growth (OD) was recorded. All cultures consumed the excess ammonium added (Figure 3). Regardless of CO_2_ source (beverage grade, PBR 1–CO_2_ or industrially reclaimed, PBR 2–CO_2_), the cultures given these gasses in excess were able to proliferate (Figure 3; PBR 1&2–CO_2_). The culture grown with air (PBR 3–Air), exhibited higher phycocyanin content when biomass was sampled. Yet the biochemical composition of other components was consistent in samples from all reactors (Figure 3).

### 3.3. Adaptation of *Cyanidioschyzon merolae* 10D to high saline culture medium using Red Sea water

**Figure 4:**
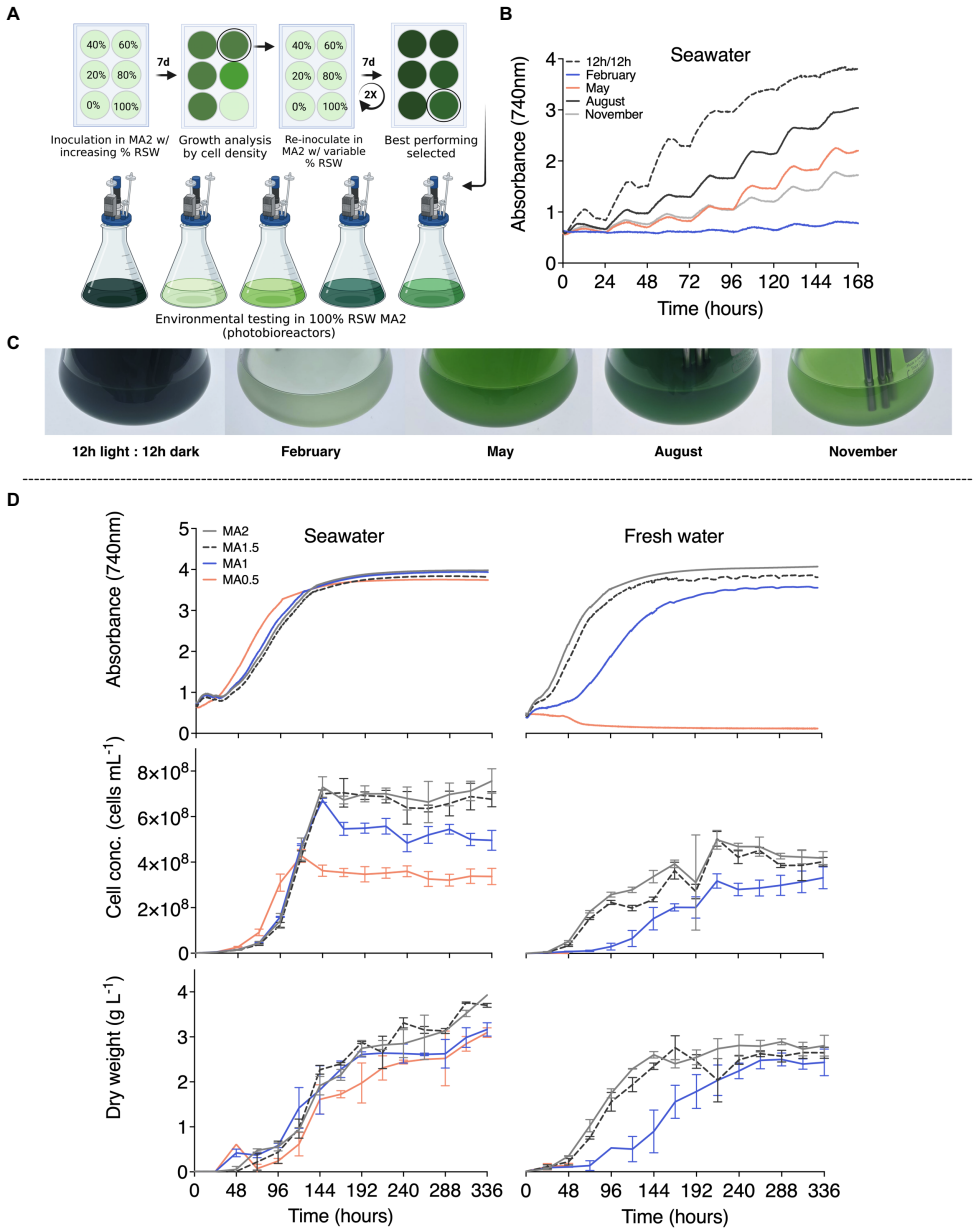
Adaptation of *C. merolae* 10D to the hypersalinity of Red Sea water. **(A)** Inoculum cultures were grown for 168 h in a range of autoclaved Red Sea water mixtures with fresh water and MA2 nutrient supplementation. At the stationary phase, the salt content condition that resulted in the highest cell density was used as inoculum for the same conditions in a new culture plate. This process was repeated for a total of 4 passes until reasonable growth was observed in 100% Red Sea water MA2 medium. This culture was used as inoculum for **(B)** photobioreactor modeled environmental testing of *C. merolae* 10D performance in temperature and light conditions for different seasons on the mid-Red Sea coast (as illustrated, upper left). Optical density (OD_740 nm_) was recorded continuously for the salt-water adapted *C. merolae* for a control culture (42°C with 12:12 h day:night illumination), modeled seasonal programs for February, May, August, and November were used to simulate winter, spring, summer, and autumn, respectively (upper right). **(C)** Culture performance was also observed photographically at the end of cultivation (pictured). **(D)** Saltwater adapted and its freshwater progenitor *C. merolae* 10D were subjected to continuous illumination and 42°C temperature conditions with culture media made using different ratios of MA2 nutrient solutions to determine the possibility of reducing nutrient inputs for its growth. Culture performance was assessed by continuous optical density monitoring in the photobioreactors, daily cell density, and biomass (dry weight) measurements over 336 h. Figure partially created with BioRender.com.

In lab-scale photoincubators, *C. merolae* 10D was cultivated in increasing mixtures of Red Sea water supplemented with MA2 nutrients (Figure 4). Inoculum was cultivated in microtiter plates and after 168 h of light and CO_2_ conditions, cells in the best growing well were used to inoculate a fresh set of dilutions and the process repeated over several weeks (Figure 4). After 4 total passes, cells proliferating in MA2 medium made with 100% Red Sea water were obtained (Figure 4). This adapted strain was then subjected to the same environmental modeling in photobioreactors as performed for its freshwater progenitor (Figure 1) to determine if salinity tolerance had affected its growth behavior (Figure 4). Based on optical density and culture appearance, the salt water adapted *C. merolae* 10D exhibited similar growth behaviors (Figures 4B,C) to cultures grown in fresh water (Figures 1B,D). The salt-water adapted *C. merolae* 10D performed best in constant 42°C and 12:12 d:n control culture conditions with Summer (August) the best seasonal performance, followed by spring (Figure 4).

Both fresh-water and the saline-adapted *C. merolae* 10D were then grown in lab-scale 400 mL photobioreactors with a constant light and temperature program, with culture medium containing full, ¾, ½, and ¼ concentrations of the nutrient solutions used in MA2 medium. Cultures were grown for 2 weeks with constant light and CO_2_ gassing. Culture performance was assessed by optical and cell densities, as well as cell dry weights (Figure 4D). It was determined that it is possible to dilute the nutrient composition of MA2 to MA0.5 in saline conditions and achieve a comparable performance of the alga in full medium (Figure 4D). Red Sea water adapted *C. merolae* 10D achieved culture dry weights of 3 g L^−1^ in 14 days even when diluted to MA0.5 (Figure 4D). Saline-adapted cultures grown with MA0.5 exhibited less cells per volume culture, but comparable biomass (Figure 4D). The optical density and biomass of saline MA0.5 cultures were comparable to, although slightly lower than, MA1.5 and MA2 counterparts (Figure 4D). However, fresh-water prepared MA2 could only be diluted to MA1 (Allen, 1959) where reduced culture performance was observed (Figure 4). Freshwater MA2 cultivated *C. merolae* 10D exhibited lower final cell densities and dry weights compared to their salt-water counterparts, although reaching comparable OD_740nm_ in a replete medium.

## 4. Discussion

### 4.1. Polyextremophilic algae for regional bioresource reuse concepts

The Arabian Peninsula is one of the more extreme environments in which human settlements have been established. Temperatures in these desert environments are consistently high, with strong irradiance and minimal precipitation (Almazroui et al., 2012; AlSarmi and Washington, 2014). These conditions are similar in most desert countries found in the so-called “hot belt” (see introduction). Despite extreme conditions, the urban population in these regions is steadily increasing, generating waste streams which require treatment and often contain nitrogen and phosphorous concentrations that can eutrophicate aquatic environments if not properly treated. This is already an issue in dense urban areas in Europe and other countries, having a direct environmental impact in water bodies that cause harmful algae blooms due to nutrient emission eutrophication process. Controlled microalgal cultivation has been identified as one of the technologies to bioremediate eutrophied industrial side-streams from aquaculture, wastewater treatment plants, and anaerobic digestion facilities (Fuentes-Grünewald et al., 2012; Mayhead et al., 2018; Silkina et al., 2019). Several reports have indicated the interest in algal cultivation in the Middle East, and at least one culture collection is established but the need for further investigation of extremophile species has been noted (Schipper et al., 2019, 2022; Hirayama et al., 2022; Rasheed et al., 2022). As the largest Middle Eastern country, Saudi Arabia could significantly advance algal cultivation using local resources (Rasheed et al., 2022). This country has many local sources of CO_2_ rich emissions, especially from industrial activities, that can be readily sourced with minimal transport distances. The combination of the high irradiance, local waste-water streams, flat non-arable land and CO_2_ sources can be synergistically combined to support algal bio-processes in Saudi Arabia and neighboring countries in the Gulf Cooperation Countries (GCC) area and act as a model for other desert locales.

**Figure 5:**
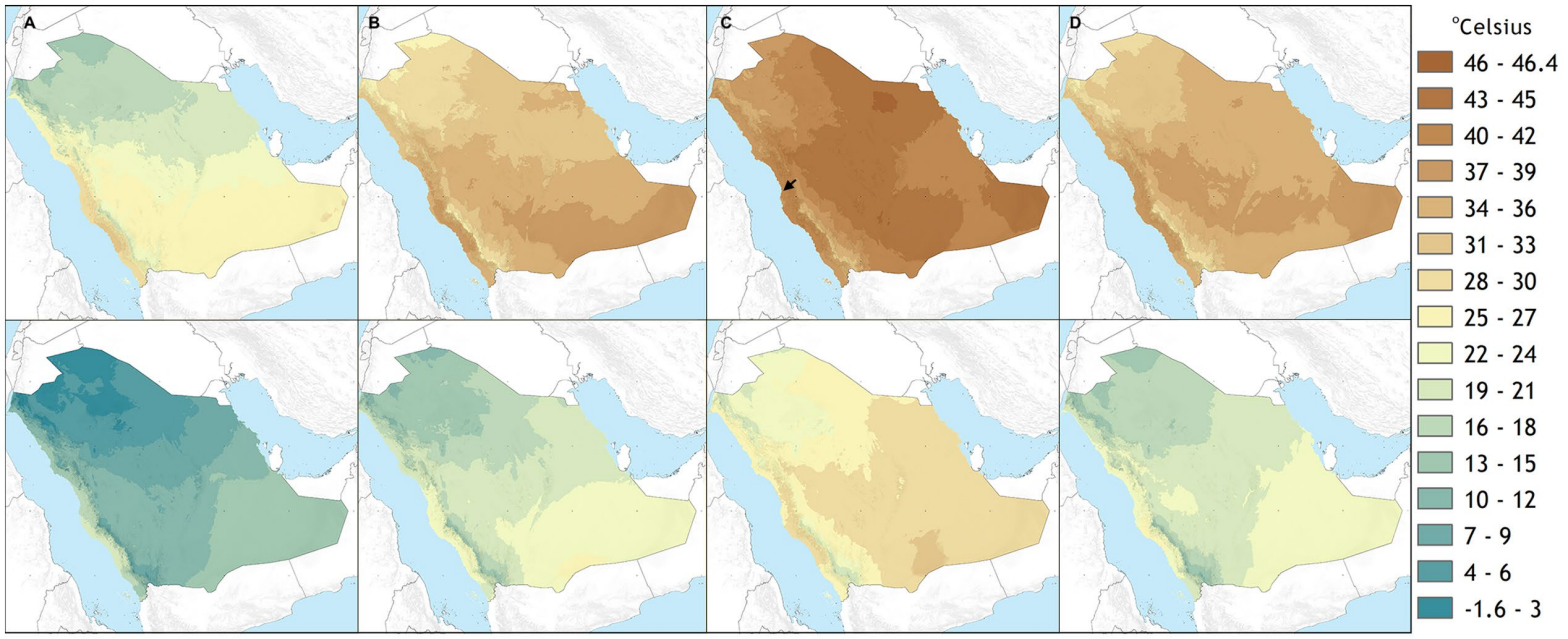
Mean maximum (upper) and minimum (lower) average monthly temperature for January **(A)**, April **(B)**, August **(C)**, and October **(D)** at ground level from 1970–2000 in Saudi Arabia (Fick and Hijmans, 2017). The location of the KAUST DAB-KSA pilot facility site of cultivation is indicated with a black arrow.

The mean maximal temperatures experienced across the Arabian Peninsula are higher than the temperate zones where microalgal cultivation is currently conducted (Figure 5; Lefers et al., 2020). The maximal summertime temperatures exceed 45°C, with regional variability depending on the site. These temperatures are above the threshold of heat stress and growth cessation of many currently cultivated algal species (Ras et al., 2013). In order to implement algal bioprocesses as part of broader resource circularity bio-economy drives, thermotolerant species are required for installations that will operate during the summer months in regions with high temperatures and light intensities (Schipper et al., 2022). In addition to thermotolerance, it has been observed that productivity outdoors in summer may not match that modeled for promising strains that perform well in indoor tests due to outer factors like UV and contaminants (Schipper et al., 2021). Given the myriad of other factors which can affect growth performance outdoors, extremophiles with culture conditions less prone to contamination, like the low pH favored by the Cyanidiophyceae, can be valuable for outdoor cultivation in these conditions.

The temperature and light simulation in lab-scale (400 mL Algem) photobioreactors used here (Figures 1, 4) was designed from atmospheric weather station data at sea level, the temperatures of which would be lower than that experienced inside a photobioreactor which would warm from the solar and infrared radiation. Growth in these tests was possible in August conditions but was not the maximal growth rate was observed in control cultures (42°C and 24 h 1,500 *μE* light). Much lower when other seasons were investigated (Figures 1, 4). *C. merolae* 10D was able to proliferate in 1000 L photobioreactors on the mid-Red Sea coast and achieve up to 2 g L^−1^ biomass at a maximal rate of 300 mg L^−1^ d^−1^ in batch mode (Figure 2). Here, it experienced temperatures ~5°C warmer than those modeled (Figure 2), much closer to its optimum at 42°C. In coastal regions of this area, high humidity moderates temperature extremes compared to inland regions. It is likely that at in-land sites in urban areas, cultures would experience significantly higher temperatures than those experienced at our study site. *C. merolae* would be an ideal candidate to proliferate in such situations and is a promising strain for phased seasonal growth concepts in such an extreme environment.

### 4.2. Acidophiles in high-strength waste-stream valorization

Outdoor cultivations conducted in this study were performed with culture medium using agricultural grade chemical fertilizers. These were found to be sufficient to enable growth of the alga outdoors at various scales. Nutrients are one of the key expenses in large scale microalgae production facilities (Fuentes-Grünewald et al., 2012). Using agricultural grade fertilizers instead of analytical grade nutrients, the reduction in production cost, and the sustainability of microalgae production has a significant effect in the reduction of operational expenditure (Singh and Das, 2014). When the culture was grown in 3-parallel 1 m^3^ tubular reactors, the cultures tolerated the addition of extra ammonium, which would cause acidic pH shifts in other algal species that require neutral pH. The cultures also tolerated either commercial beverage-grade or industrial emission reclaimed “green” CO_2_ gas sources (Figure 3; PBR 1 + 2 – CO_2_, respectively). Cells grown with either CO_2_ source did not differ in biomass composition containing equivalent phycocyanin (PC), protein, carbohydrate, and lipid contents (PBR 1 + 2, Figure 3). The culture which was starved of carbon, only atmospheric CO_2_ levels, exhibited higher PC concentrations at 72 h (PBR 3–Air; Figure 3). This could be a means of increasing PC content prior to harvest, but needs further investigation. Experiments which could be conducted to explore this phenomenon would be similar to those done for increasing phycocyanin content in cyanobacteria or lipid accumulation in algae, such as 2-stage cultivation modes, or variations on light intensity (Fuentes-Grünewald et al., 2012; García-López et al., 2020). The PC of *C. merolae* is considered more thermostable than that of other currently harvested organisms such as *Arthrospira platensis* and could be a valuable co-product from the biomass (Rahman et al., 2017). These behaviors suggest *C. merolae* 10D as an acidophile is highly suited to industrial waste re-valorization processes which could convert high-strength ammonia containing wastewaters and industrial CO_2_ sources into valuable biomass. We suggest this promising extremophile as a unique strain to be used in large scale production facilities for CO_2_ reuse applications.

### 4.3. Opportunities for Cyanidiophyceae biotechnology in a regional context

Extremes of heat in summer are balanced by moderate and even low temperatures in the winter and spring months in the Arabian Peninsula (Figure 5). Photobioreactor seasonal simulation and growth performance of *C. merolae* 10D in these months indicated that the polyextremophile did not perform optimally under lower temperature regimes (Figures 1, 4). From a bioprocess standpoint, engineering in-culture heating in these off-months is technically straightforward as heating processes require less energy than cooling. Culture heating can be achieved, for example, with industrial waste heat recirculation (Ekendahl et al., 2018). It should be possible, therefore, to cultivate *C. merolae* 10D year-round in this environment, so long as bio-processes are designed with relevant parameters and approaches in mind.

The cultivation in acidic conditions requires some considerations in the source of inputs for scaled *C. merolae* 10D cultivations. CO_2_ injections into the culture medium (effluent inputs) could be used to reduce pH of solutions depending on alkalinity. High-strength ammonia containing waste-waters like those from aquaculture will also be appropriate for *Cyanidioschyzon merolae* 10D cultivation as the consumption of ammonium can further drive the culture to acidic conditions (Henkanatte-Gedera et al., 2015; Selvaratnam et al., 2016; Nirmalakhandan et al., 2019). This additional acidification of input waters by CO_2_ may enhance the potential of *C. merolae* as a vehicle for carbon reuse, valorization, and circularity. *C. merolae* 10D cultivation could be best performed on already acidic waste streams, like those of the dairy industry. Process designs with this organism will have to determine the best ways to incorporate its acidic cultivation conditions.

The ability to adapt *C. merolae* to saline cultivation conditions (Figure 4), also opens the possibility for cultivation in sea waters sourced along the coastlines of desert countries, improving the sustainability of commercial large scale microalgae facilities (Ishika et al., 2017; Panagopoulos and Haralambous, 2020; Merlo et al., 2021). We observed that saline adapted cultures could achieve similar growth with lower nutrient inputs, likely indicating osmotic balance in fresh-water conditions is maintained from high amounts of dissolved nutrients. The native habitat of *C. merolae*, besides acidic hot-springs (Toda et al., 1995; Matsuzaki et al., 2004), is a high mineral containing environment which could suggest why attempts to cultivate in fresh waters have required higher ammonium and phosporous titers than other algae (Minoda et al., 2004). High-strength aquaculture effluents with CO_2_ injections may be used as inputs for culture of saline adapted *C. merolae* 10D. Further efforts with adaptive laboratory evolution, may also elicit new phenotypes and a higher accumulation of target products, as has been shown in saline *Schizochytrium* sp. (Sun et al., 2018). *C. merolae* 10D has been shown amenable to genetic engineering by target homologous recombination in its nuclear genome, opening the capacity for metabolically engineering increased product profiles from its biomass (Fujiwara et al., 2013; Miyagishima and Tanaka, 2021). Recently, we demonstrated that expression of green algal beta carotene ketolase and hydroxylase from the nuclear genome of *C. merolae* 10D and the targeting of the recombinant protein products to the algal plastid could generate ketocarotenoids canthaxanthin and astaxanthin in this red alga (Seger et al., 2023).

Saline adapted, wild-type or engineered, *C. merolae* 10D could be valuable as a microorganism for nitrogen and phosphorous removal from on-land marine aquaculture concepts, while generating a protein-rich biomass that can be added to feeds, toward more efficient circular use of bio-resources (Figures 3, 4). Biomass observed in outdoor cultivations maintained a high protein content close to 50% of its biomass, with carbohydrates representing only ~10% and total lipids ~20% (Figure 3F). As *C. merolae* is a cell-wall deficient species, its rupture and incorporation into feed as a protein biomass is straightforward and requires little energy inputs during downstream processing (De Luca et al., 1978; Kuroiwa et al., 2017; Miyagishima and Tanaka, 2021). This property also allows simple extraction of its thermostable phycocyanin or its biomass could be used to make bio-stimulant fertilizers due to its high protein content, for emerging contained environment agriculture concepts suitable for this region (Lefers et al., 2020). Engineered biomass containing ketocarotenoids, may also be of increased value in fish feeds, where astaxanthin is used to enhance fish color, however, this will require further market considerations and regulatory approvals (Ambati et al., 2014).

## 5. Conclusion

Here, we demonstrate that the polyextremophile *C. merolae* 10D is a promising candidate for algal-based resource circularity in hot desert environments. Its thermotolerance allows its cultivation even in the extremes of desert summers and its acidic preferences can be used to minimize contamination and maximize ammonia removal from liquid and CO_2_ waste streams. The work reports scaled cultivation of C. *merolae* in the summer months in Saudi Arabia and shows that it can be adapted to salinities at least as high as those observed in Red Sea waters. C. *merolae* could be an interesting candidate for on-land marine aquaculture wastewater treatment and revalorization in addition to the reuse of high CO_2_ concentration emissions. This is the first report of the scaled cultivation of *C. merolae* 10D and the first demonstration of its growth in the Middle Eastern context. Our report sets a foundation for increasing investigations into the use of *C. merolae* and its biomass for bioresource circularity and applications like feed, fertilizer, and other high value bio-products such as thermostable phycocyanin. This report indicates that *C. merolae* 10D may hold promise for biotechnological application using the resources found in abundance in the Arabian Peninsula and other desert regions such as North African countries, Chile, and Northern Australia with urban and industrial development.

## Data availability statement

The original contributions presented in the study are included in the article/Supplementary material, further inquiries can be directed to the corresponding authors.

## Author contributions

MV-V was responsible for cultivation of *Cyanidioschyzon merolae* 10D in lab-scale, adaptation to sea water, lab-scale photobioreactor operation, sampling, data analysis, and figure preparation. BF was responsible for lab-scale bioreactor operation, sampling, data analysis, and figure preparation. RG-P and GR-V were responsible for the outdoor cultivation of *C. merolae* from 5 L-1000 L. RK and RM were responsible for biochemical characterization, data interpretation, and data reporting and analysis. CF-G and KL were responsible for project design, funding acquisition, data analysis, and manuscript writing. All authors contributed to the writing of this manuscript and figure layout decisions.

## Funding

The research reported in this publication was supported by KAUST baseline funding awarded to KL and the DAB-KSA project (number: 52000003916) funded by the Saudi Ministry of Environment Water and Agriculture (MEWA) through Beacon Development (KAUST).

## Conflict of interest

The authors declare that the research was conducted in the absence of any commercial or financial relationships that could be construed as a potential conflict of interest.

## Publisher’s note

All claims expressed in this article are solely those of the authors and do not necessarily represent those of their affiliated organizations, or those of the publisher, the editors and the reviewers. Any product that may be evaluated in this article, or claim that may be made by its manufacturer, is not guaranteed or endorsed by the publisher.
